# The Nursing Supplement

**Published:** 1889-10-12

**Authors:** 


*The Hospital,*
OCTOBER 12, 1889.

*Extra Supplement*

### The Nursing Mirror.

All Contributions for this Supplement should be addressed to the Editor, THE HOSPITAL, 139-140, Salisbury Court, Fleet Street, London, E.C., and should have the word "Nursing" plainly written in left-hand top corner of the envelope.

### En Passant.

BOURNEMOUTH NEWS.—The Nurses' Institute at Bournemouth has dissolved, and the Victoria Home does not supply so many nurses as are needed. The question, therefore, has come forward, is there to be another institute started, or will the Victoria Home enlarge its operations? The one home ought to be enough for Bournemouth, but the nurses of the old institute have some objection to becoming members of the Victoria Home. After considerable discussion, a committee has been appointed to organise a new Bournemouth Nurses' Institute. Dr. Scott is one of the committee.

VILLAGE NURSING.—A letter lately appeared in the *Times*, signed by the Hon. Mrs. Dundas, of Leyburn, asking help for a "Village Nursing Association." Two or three points in the letter were not too practical, and we were glad to see an answer from a correspondent, who wrote thus: "I anticipate that the opinion of the medical profession will be that the training of such nurses should be nearer two years than six or twelve months, for it must not be lost sight of that the responsibilities of a village nurse are greater than a town nurse. I confess I am not cognisant with the working of the fund raised by the women of England in commemoration of the Queen s jubilee, and which her Majesty, with her never-failing thoughtfulness, so graciously and handsomely devoted for the very object (as I understood) of providing in large towns exactly what the Village Nursing Association is now being formed to do in the rural districts; but results do not appear in a day, although I feel sure, considering the able hands in which the Queen's funds were placed, that no time is being lost in organising and carrying on the work entrusted to their care. I cannot but think that there is great danger, for very many reasons, of multiplying associations for carrying on the same work."

MARY ADELAIDE LETTER.—The last quarterly letter to the Mary Adelaide nurses deals with Indian hospitals, and gives an interesting account of the start of the Canning Home for Nurses: "At Calcutta I went to the European General Hospital, to which is attached the Canning Home for Nurses, established as a memorial of Countess Canning, whose husband, Earl Canning, was Viceroy of India about thirty years ago. This is under the charge of sisters from the Sisterhood of St. John Baptist, at Clewer, near Windsor, who, it is almost needless to say, do their work admirably, and are able by means of the Canning Home to train a regular supply of probationers for hospital and private nursing. This home is an invaluable institution, and before Lady Canning's time nothing of the sort existed in India. She had been all her life interested in nursing and many other works of charity, and when she went to India she soon turned her attention to the necessity for providing a regular supply of properly trained nurses for the European patients in Calcutta. After her death, which occurred there in 1861, it was felt by the many friends and relations who had deeply loved and admired her that no fitter memorial of so good and noble a life could be suggested than the establishment of a home and training school for nurses in connection with one of the large hospitals of Calcutta, and when sufficient funds were collected the Canning Home for Nurses was started."

SECESSION TO ROME.—Miss Henrietta Madden, after long service as a Protestant deaconess of the Protestant Hospital in connection with the North Dublin Union, has seceded to the Roman Catholic Church, where she will probably be given work in some nursing sisterhood. Miss Madden is a sister of the present Solicitor-General for Ireland, and a great favourite at the hospital where she worked so long.

SHORT ITEMS.—Nurses can easily obtain employment at the Metropolitan Asylums Board Fever Hospital just now.—Dr. T. Parvin, of Philadelphia, has published a book on "Obstetric Nursing."—Lieutenant Tottenham, while a patient at Devonport Royal Hospital, broke from his nurse in a fit of delirium, threw himself out of the window, and was killed.—Two more nurses at Birmingham have caught scarlet fever; those who were first ill are all recovering.—At the annual meeting of Lowestoft Hospital, Miss Hunter (matron) and the nurses were congratulated on their excellent work.—Medical training will soon be supplied to women at Glasgow.—Sister Mary, of Guy's Hospital, is about to start a nursing institution at Biarritz.

NURSES IN AUSTRALIA.—The annual report of the Alfred Hospital, Melbourne, has just been issued, and contains a reference to the coolness of the nurses at the late fire. It says: "A disastrous fire occurred on 26th December last, by which the whole of the upper portion of the west pavilion was destroyed, and the lower rendered perfectly useless until the structure was reinstated. Happily no lives were lost, nor could it be affirmed that any ill effects to the patients followed the fire. This result was due, in the first place, to the coolness and intrepidity of the nurses and other members of the staff, and, in the second place, to the timely aid received from the defence force, who gratuitously placed at the disposal of the managers a number of tents, and lent their services to erect the same. During the past year four Class 'A' and seventeen Class 'B' pupils have entered the school; while, on the other hand, fifteen having passed the necessary examinations, and obtained their certificates of competency, have left the institution. The present number of pupil nurses on the hospital staff is twenty-eight. Only ten students (seven ladies and three gentlemen) have entered for medical practice during the current year. This diminished number was due in great measure to the want of conveniences for students at the hospital, a difficulty which, from the absence of the necessary funds, the managers have not yet been able to overcome. Nearly a year ago the university authorities asked if instruction could be afforded students in such special branches as diseases of women, of children, and of the eye, ear, and throat. To this communication the managers could only reply that, though they were desirous of meeting the requirements of the university, the present accommodation at the hospital was totally inadequate for the purpose. The maintenance account, after allowing for assets and liabilities, was exhausted; while the building fund, although it shows a credit balance of £3,000, has liabilities of over £1,000 to meet. It would thus be seen that with the present insufficient accommodation for patients, nurses, and students, the revenue—though in excess of last year's—had barely sufficed to meet the expenditure. The number of patients under treatment during the year 1888-9 was as follows: In-patients, 1,612; out-patients, 2,577; casualities, 648; total, 4,837."

### Case Taking.

THIRD PAPER.

PERHAPS the best method of teaching case taking is to give some specimen cases taken by nurses. We have luckily got some of these on hand, and we propose to take first a very simple case. Special cases of rare diseases can be found in the medical journals, but are not so useful, and far too elaborate for our purpose.

*Name*, Alice Smith; *age*, eight months; *sex*, female. *Physician*, Mr. Jones; *case*, enteritis; *admitted*, June 8. *Family history*: Father and mother both alive and well. Patient is their first and only child. *History of present illness:* Mother says that patient has been ill since June 5; severe diarrhœa and vomiting. Three weeks ago, the patient had convulsions. Since Friday, patient has steadily got weaker. *Patient's condition*: The child is pale and emaciated, fretful and hungry; temperature 100, pulse 108, motions six. *Treatment*: Hyd. cretâ gr. II., pulv. rhei. gr. III., ft. pulv. bis. die.; infant's diet, and 10 drops of brandy every hour. *June 14.*—Patient improving. Temp. 99·2, pulse 106; diarrhœa has practically ceased the last two days. *June 20.*—Pulv. rhei. gr. II., sodae bicarb. gr. II., bis. die. Lime water. Patient increases in weight and strength. Temperature normal. *June 24.*—Patient discharged, cured.

The above are the notes of a very simple case taken in a children's hospital. The notes of such a case are easily kept, and, indeed, in a medical ward the case taking is always less troublesome than in a surgical ward. The following notes of a surgical case were taken in a workhouse infirmary: *Name*, Jane Evans; *age*, 34; *sex*, female; *surgeon*, Mr. Howel; *case*, Genu Valgum; *admitted*, Aug. 10. *Family history*: Father and mother alive and well, two sisters also alive, none of the rest of the family afflicted like patient. *Patient's history*: At the age of 11 years patient had rheumatic fever consequent upon getting wet in rain, fever lasted three months, but patient seems to remember but little about it. When convalescent she was terribly crippled, could not walk more than a few steps, and her fingers were drawn towards the palms of her hand. Six months after a red swelling appeared below great trochanter of left femur; an abscess formed, which lasted three years. Several splinters of bone worked out through abscess. Four other abscesses broke out during subsequent years; one on the left thigh, two on the right ankle, and one just below right knee. These abscesses were dressed with various ointments recommended by friends, and with "swelt rags," by which the patient seems to mean lard spread on old linen. *Present condition*: Patient is thin, but healthy; no abscesses now, and the hands are no longer deformed. She walks very clumsily. Lucas crossed leg progression; she feels her deformity painfully. It also prevents her obtaining a situation, and has brought her to the workhouse. *August 13.*—Dr. Howel has got patient admitted at Guy's Hospital. *August 18.*—Dr. Howel tells me he was present at Guy's today when Jane Evans underwent operation: osteotomy of one leg; tenotomy of other. *September 30.*—Jane Evans came to see me to-day. She walks on crutches, but her feet are now straight, and the surgeons tell her she will shortly be able to walk properly without aid.

Here is the history of a case of mortus coxæ in a boy aged six. "Patient received a blow on left knee from a stone two weeks ago. No notice was taken for three days; then he was observed to limp. The parish doctor was called in, and advised rest. When admitted to-day the patient was suffering great pain in knee, and was unable to walk. The left leg was wrapped in cotton wool, the knee was swollen and inflamed, and painful when moved. The thigh and knee flexed, and the foot exerted; slight lordosis. Extension of 2lb. applied."

It will be observed none of the above cases are quite full, nor answer all the requirements of case taking we have noted; still we have thought it best to give genuine cases taken by nurses, choosing the best that have fallen into our hands.

### "The Bitter Cry of 'They.'"

BY ONE OF THEM.

(*Dedicated to Modern Doctors.*)

WE are devoted to originality, and above all things we despise sameness, tameness, and mediocrity. We consider ourself unique—(Excuse, kind and lenient reader, the apparent paradox, for the journalistic plural is too impressive to be lightly parted with.)—we consider ourself unique, peculiar (in a pleasant sense, of course), and possessed of a delightfully fresh, unconventional nature. No deeper humiliation can be contemplated by us than the being treated of, thought of, spoken of as a "class," like Editha's burglar, or any other such inferior organism.

We consider it as gross an insult to be relegated to classes as we should to be esteemed a part of the "masses." Other people may be—probably are—made in one pattern, but we have always deemed ourself erected upon a hillock of individuality from whence the dead level of everybody else can be looked down upon with good-humoured toleration. We, in fact—to use a vulgar phrase of commercial extraction—are "the only one in stock." We are not, however, conceited. We do not esteem ourself invariably "the best." It is enough that we are "the" something or anything, a dignified isolated definite "The," not a mild, conventional "a" in a crowd of "sames."

This being the case, why, O ye awful doctors, do ye assault our exclusive, personal eminence, and seek with your levelling theories to debase us to the flat and dreary plane whereon are ranged the ranks of your ready-made battalions? Why challenge our right to—even our possession of—one single spark of originality, rend from us every cherished shred of egoism, and strive to involve the calm unapproachable "We" in the distracting hubbub of "Them"? Of course, there are certain wide general rules of physical construction which we are willing to acknowledge. We bow to the vulgar necessity of eating and drinking when hungry and thirsty, sleeping when tired, etc. We submit to allow tongue, pulse, and temperature (even our own) to be within the range of the physician's province, and hence liable to be relegated to groups and classes. Exquisitely painful though it be to our inner consciousness, we protest not when told that our symptoms are the "ordinary" ones for measles, mumps, whooping-cough, etc. This we are prepared for, and can endure with philosophy. But there is a point beyond which we would fain dispute the medical sway. Our intellect, our tastes, our propensities—in a word our character—should be our own; and yet these are in danger of being wrested from us, and becoming, in the ruthless hands of Æsculapius, mere classifying signs. So philosophical and psychological, etc., has the medical art become, that, in lieu of the harmless necessary questions of our youth—Is your appetite good? Does your head ache? Do you suffer from cold feet, etc.?—we are greeted with such astounding enquiries as—Are you fond of Shakspeare? Do you prefer Bach to Wagner? Do you take an interest in politics? Are you a believer in Mr. Gladstone? Are you fond of the theatre? Do you prefer modern art to the old masters? Do you read novels, history, poetry? Can you understand Browning? etc. And, as if these questions were not sufficiently irritating to one's sense of moral privacy, each reply is greeted with the rejoinder, "Exactly, yes—so they all say—that is what I expected—they all do. Yes, just like the rest of them"—so crushingly destructive of one's cherished feeling of removedness from the general herd.

And as soon as all our most distinctive, and (as we fondly imagine) original sentiments have been dragged to the surface, we perceive, to our unmitigated discomfiture, that our medical catechiser is proceeding to diagnose our case from these "symptoms" which, he cheerfully assures us, are common to "all of them," and we are informed that we henceforth appertain to the class of dyspeptics or neurotics, etc. Instructions follow as to the system of diet which "they" always find beneficial, the amount of exercise "they" all take, the intellectual pursuits which do most obtain amongst "them," the follies which "they" are most prone to; and, in fine, life opens out before us no longer with individual tastes and occupations, but beset at every turn by the other members of our "class"—our *doppel-gangers*—who do our deeds, think our thoughts, opine our opinions, until, to our fevered imagination, existence becomes strangely like unto that awful game of our childhood—"Neighbour, O neighbour, I come to torment you . . . to do so-and-so as I do."

There still remains a consoling doubt as to whether we shall ever in reality meet with—or if meeting, recognise—these irritating fellow-sufferers. But, alas! our confidence in ourself has been rudely shaken. Our delightfully fresh impulses, our original imaginings, our unique tastes—where are they? Jotted down in Dr. ——'s note-book as incontrovertible signs of our involuntary adherence to some common-place class, whose members are colloquially and professionally known as "They."

### Everybody's Opinion.

[*Correspondence on all subjects is invited, but we cannot in any way be responsible for the opinions expressed by our correspondents. No communications can be entertained if the name and address of the correspondent is not given, or unless one side of the paper only be written on.*]

### SUPERANNUATED AT FORTY-THREE.

Not Wanting Work writes: I have been much pained by reading the cases of Nurses E. and E. M. Smith, and am sorry to find that there are many others in the same position. I am a trained nurse (not 40) with 15 years' experience. I am convinced by contact with the public that nurses of 40 or over 40 are not rejected by them, but, on the contrary, often sought for and not found. Why? Because of the sweating system so largely practised in the nursing world. Nurses over 35 are pushed out of work by the sweaters, who are the principals of a large number of the so-called nurses' homes. These women will not engage experienced nurses while they can get young nurses just leaving hospital for small salaries. The suggestion of "A Hospital Governor" is a timely one, and one that could be carried into practice in every town where a nurses' home is required. The co-operation of the doctors is the first thing to be obtained. There are many places where two or three sweaters have nurses' homes, and thus a large portion of nurses' earnings are spent in house rents and other things, where as one home under the superintendence of a salaried matron would supply the wants of the place.

### NURSING IN WORKHOUSES.

Ex-Nurse writes: Periodical complaints of the dulness of a nurse's life in a union infirmary, and of the bad nursing there, lead me to offer my experiences, some years ago, as charge-nurse at Chorlton Union Hospital. It is always well to see both sides of a question. The untiring zeal of the resident medical officers, and the skill and devotion of their own trained nurses in active cases, far surpassed anything I had ever seen during my three years' previous experience at one of the largest hospitals in the North of England. The nurses were not socially inferior, and their skill, either in the medical or surgical department, would compare favourably with that of their hospital brethren, or sisters, I should say. Indeed, firmly believing in that old adage which says "practice makes perfect," I think, given the same variety of work, they would carry away the palm. There is a proficiency and thoroughness about them which the hospital nurse has no chance of acquiring, as the "dressers" do all the bandaging and dressing of wounds, which falls to the lot of the union hospital nurse. I have seen over and over again in the above-mentioned hospital acute cases of pneumonia successfully cured, which could be attributed by an impartial eye to nothing else than clever nursing, and I have no hesitation in saying that the same cases would not have had the same chance of recovery in a hospital. I have known of many deaths from pneumonia in a hospital, and I only know of two, out of a larger number of cases, in the union hospital. I consider the latter patients, whose frames are debilitated and wasted by want and exposure, require more care, and their recovery is consequently more miraculous. I also know of the amputation of the breast of an old lady over 70 years of age who made rapid progress, and was completely cured; also the case of a man who had been told at the hospital they could do nothing more for him, whose leg was amputated at the union hospital, and he, who was once so attenuated as to be the shadow of death, is now fat and prosperous. There are other cases which I could name. These at present will suffice, and will, I think, prove conclusively that at least in one union hospital the nursing staff was not inefficient. I believe the reason why a union hospital patient has a better chance of recovery is because he is attended by one nurse who is responsible for him, and who knows without a lot of red tapeism when the last poultice was applied and when to apply the next; and you know, sir, as well as I do, that "too many cooks proverbially spoil the broth."

### ASYLUM ATTENDANTS.

J. B. writes: I have read with great interest the letters respecting asylum attendants, and quite agree with your correspondents that they should receive a training both mentally and surgically. The first and only way to remedy the ignorance existing amongst asylum attendants is to have only hospital-trained matrons and head attendants, whereas the majority of the latter are only very inexperienced, some having no training, and, in fact, are made charge or head attendants after six months' experience, and perhaps never been in an asylum before. I have never received any information from doctor or matron on mental or surgical cases. Another reason why only hospital-trained matrons and head attendants should be engaged is, in cases such as confinements what does an inexperienced girl know of midwifery? and lunatics have the same feelings as a sane person and require as careful nursing. There are many improvements to be made before our county asylums will ever attain anything approaching to perfection. I have also noticed that the penny novelettes are read by a great many attendants, but there is a remedy—viz., establish a library, and the penny rubbish will be no longer a necessity; or better still, establish a class, say three times a week, and give lectures on what is necessary to an asylum attendant.

### NURSE OR DOCTOR.

Nurse Davies writes: I should imagine from what Dr. Richardson says in his book "On the Duties and Conduct of Private Nurses," that he had a spite against one. He says the "drunken Sairey, at most, left undone her work, whereas the presumptuous nurse does active mischief." Now, I maintain that no thoroughly good trained nurse, I mean an educated woman (lady or otherwise), would be presumptuous or do any active mischief; she would carry out the doctor's orders to the letter. But does Dr. Richardson know how hard this is in some cases, especially where the patient or friends object to the line of treatment, and think they know more than all the doctors and nurses put together? It would be far better if doctors would look more to the comfort of nurses working for them, especially to the amount of sleep they get, which is of vital importance. People that pay thirty shillings or two guineas per week for a nurse naturally like to have their money's worth, and seem to think we can do any amount of work without rest, and are quite surprised when tiredness is complained of. I should like to administer a hint to the public in general, and inform them that we nurses are not machines wound up by the day or week, warranted to go.

### Examination Questions.

### A NEW COMPETITION.

In deference to the wishes of our readers, we will open for the winter months a new competition for nurses only. A question on nursing will be asked every month, and the best answer will be printed in our columns, and the writer will receive a book. The question for October is: "State what you know of the antiseptic treatment of wounds." The answers must be short, concise, and clear, and written on one side of the paper only. They must be addressed to our office at Salisbury-court, the word "Nursing" being written in left-hand top corner of envelope. They must reach the office by October 23. Each answer must be accompanied by the writer's full name and address.

### Appointments.

Great Northern Central Hospital.—Mr. Philip R. W. Santi, F.R.C.S.Eng., has been appointed house surgeon to this institution in the room of Mr. C. Mortlock, M.R.C.S., whose term of office has expired.

Victoria Infirmary, Glasgow.—At a special meeting of the governors, held on Oct. 1, Dr. Donald J. Mackintosh was unanimously elected superintendent and resident medical officer, and Miss Ross as matron. Dr. Mackintosh was for two years resident surgeon at Glasgow Eye Infirmary, and for the last three years senior assistant physician at the Belvidere Hospital, Glasgow. Miss Annie Ross, of the Royal Infirmary, Glasgow, has been appointed first matron of the new Victoria Infirmary. She was one of 41 applicants for the post. Miss Ross was trained at Guy's Hospital, London, and afterwards took charge as Sister of Bright Ward. She has since been nearly four years superintendent of nurses in the Royal, and has always acted for the matron in her absence. She is a great favourite amongst her fellow nurses, and holds excellent testimonials. Miss Ross joined the National Pension Fund for Nurses at an early period, as she cordially approved of its objects.

### Notes and Queries.

To Correspondents.—1. Questions may be written on post-cards. 2. Advertisements in disguise are inadmissible. 3. In answering a query please quote the number. 4. If a private answer is desired, a stamped addressed envelope must be forwarded.

### Queries.

(95) *Nursing the Lepers.*—Kindly inform me to whom I should apply for nursing work amongst the lepers. *B.*

(96) *Old Work as New.*—Can you tell me if the articles which recently appeared in a contemporary, "A Guide to Medical and Surgical Nursing," ever appeared before? They seem familiar to me? *Annie.*

(97) *Hoblyn's Dictionary.*—Please tell me the price. *Nurse Middlesbro.*

(98) *Certificates.*—Nurse G. wishes to know how she can obtain a certificate of nursing. She trained at Bristol, where they were not given.

(99) *Nursing Lectures.*—I have lately attended some excellent lectures on nursing. How can we persuade the lecturer to publish her instructions? *Nurse Edith.*

(100) *Doll Competition.*—Particulars wanted. *Metropolitan Nurse.*

### Answers.

(5) *Home for Epileptic Children.*—A correspondent writes to ask if there is any home into which a little boy, aged ten, subject to epileptic fits, can be received. The child's parents are poor, but as he is becoming troublesome to manage, they would pay a small weekly sum towards his support.

(6) *Home for Epileptic Girls.*—A district nurse is anxious to hear of an institution to which she can send her daughter, who is epileptic, and is obliged to live at home in consequence. When well, she is a good needlewoman, but periodical fits prevent her living alone.

(77) *Massage.*—Some time since one of your correspondents asked if Dr. Metzger took pupils in massage. I believe not now, but his system can be learnt from Fraulein Muller, 8, Upper George-street, Bryanston-square, W. *Matron.*

(93) *Beef Tea.*—Nurse Davies should know that reason and right feeling both demand that in each individual case the proper person to decide what kind of beef tea is to be given is the doctor in attendance. Whenever there is the least doubt the attendant physician alone is to be consulted, and his decision must be final.

(95) *Nursing the Lepers.*—Apply to Mr. Carlile, Church Army Nurses Home, Edgware-road, W.

(96) *Old Work as New.*—Yes, the articles are merely a reprint of a book called "Hints to Nurses," published by Maclachan and Steward in 1877, since which date *we* thought nursing knowledge had increased.

(97) *Hoblyn's Dictionary.*—Price 10s. 6d., unless you get it second-hand. See answer to query by *M. B.* last week.

(98) *Certificates.*—The quickest way is to train for a year at Burton-on-Trent or Stafford Infirmary. They give certificates at the end of a year. Also Salop Infirmary.

(99) *Nursing Lectures.*—Send us the name and address of the lecturer, and let us try our powers of persuasion.

(100) *Doll Competition.*—Particulars were given last week, and in our back numbers. Dolls can be dressed singly or in groups, and must be sent to the Editor by October 31st. To each doll must be affixed the name and address of the exhibitor and dresser. There is no entrance fee. £10 will be given in prizes for the best dressed dolls.

*Nurse B.*—We never give directions about treatment.

*A. B.*—We never suggest remedies. Consult your own physician.

*Certificated Midwife.*—The Midwives' Institute, 15, Buckingham-street, Strand, of which Mrs. Nicholl is secretary, have in preparation a Bill of which notice was given in Parliament last session. You should join the Midwives' Institute.

*Specialists.*—Procure the *Hospital Annual* and see list of staff of different hospitals for nervous diseases. We do not recommend specialists or medical men.

*Asylum Attendant.*—We published a series of lectures on "The Nursing and Management of the Insane," by T. Duncan Greenlees, M.B., during April and May. Write to the publisher for our May and June monthly numbers.

*Would-be Masseuse.*—If you desire private lessons, apply to Mrs. Creighton Hale, 46, Maddox-street, London.

### Vacancies.

The following vacancies are announced:

### Matrons.

Royal Hospital for Children and Women, £60.

York Lunatic Asylum, £60.

### Assistant Matron.

Norfolk and Norwich Hospital, no salary.

### Nurses.

Arbroath Infirmary, £20.
Aylesbury Infirmary, £25.
Bath Mineral Hospital (Masseuse).
Bournemouth Nursing Institute, £25.
Bridgewater Infirmary.
Brighton Fever Hospital, £28.
Cardington Children's Hospital.
Carmarthen Infirmary, £23.
City of London Hospital, £20.
Highbury Institute, £20.
Hull Sanatorium (Fever), £30.
Jersey Nurses' Institute (two), £25.
Kent Nursing Institution.
Leicester Fever Hospital.
Levick Institution, Paris (several).
Miller Hospital, Greenwich, £22.
Mitchell Home, Torquay (two), £22.
Newport County Infirmary, £25.
Nurses' Home, Hull, £25.
Nurses' Home, Newcastle (six).
Nursing Institution, 96, 97, 98 Wimpole-street, London, W., Mr Wilson has several vacancies for thoroughly trained nurses.
South-Eastern Fever Hospital, £30.
Southport Nurses' Home, £20.
South-Western Fever Hospital, £22.
St. George's Home, Sheffield.
St. Helena Home, St. John's Wood.
St. John's Institute, Southport.
Stoke-on-Trent Nurses' Home (several), £25.
Victoria Home, Bournemouth, £25.
Waifs and Strays Society, £20.

### District Nurses.

Bedford Institute, £26.
Blackheath Institute, £25.
Frome Nurses' Home.
Hitchin Nurses' Institute, £23.
Kemerton Rectory (midwife).
Woodlands, Glasbury (midwife).

### Probationers.

Halifax Fever Hospital, £14.
Her Majesty's Hospital, Stepney.
Hull Royal Infirmary.
Nurses' Home, Sheffield.

### Amusements and Relaxation.

SPECIAL NOTICE.—ACROSTIC COMPETITION.

The Prize Editor regrets that he is unable to start the Acrostic Competition as too few names have been sent in to make it possible to offer a prize.

**N.B. Third Quarterly Word Competition Commenced October 5th, 1889, ends December 28th, 1889.**

Three Prizes of 15s., 10s., 5s., will be given for the largest number of words derived from the words set for dissection.

Proper names, abbreviations, foreign words, words of less than four letters, and repetitions are barred; plurals, and past and present participles of verbs, are allowed. Nuttall's *Standard* dictionary only to be used.

N.B.—Word dissections must be sent in WEEKLY, arranged alphabetically, with correct total affixed.

The word for dissection for this, the *Second* week of the quarter, being "PARTRIDGES."

### Results of Thirteenth Week.

| Names. | Oct. 3rd. | Totals. | Names. | Oct. 3rd. | Totals. |
|---|---|---|---|---|---|
| Nurse Duty | 7 | 1018 | Percy Verance | — | 18 |
| Jumps | — | 30 | Gleniffer | — | 175 |
| N'importe Qui | 6 | 804 | Gypsye | 6 | 915 |
| Tinie | — | 186 | Ladyr | — | 127 |
| Amicus | — | 430 | A. B. | — | 170 |
| Patience | 7 | 1027 | Essie | — | 393 |
| Broxbourne | — | 398 | Cowboy Bill | — | 179 |
| Speedwell | 7 | 901 | M. L. A. | — | 117 |
| Lightowlers | — | 356 | W. G. R. | 7 | 785 |
| Lauriston | — | 345 | Little Tuppy | — | 259 |
| Sister | 7 | 931 | Embryo | — | 56 |
| Rattler | — | 28 | Jenny Wren | 7 | 758 |
| Paignton | — | 334 | Pearl | — | 132 |
| Fassifern | — | 141 | Condorcet | — | 450 |
| W. R. E. | 7 | 997 | R. W. | — | 43 |
| Buxton | — | 167 | Electrician | 7 | 767 |
| Nux Vom | — | 263 | Nurse Amie | — | 89 |
| Esperance | 6 | 1001 | Sunshine | 7 | 666 |
| Spes | — | 24 | Juvenis | — | 23 |
| Secnarf | — | 229 | Qu'appelle | — | 800 |
| M. W. | 6 | 1001 | K. Jarman | — | 43 |
| Rover | — | 226 | Reveal | — | 196 |
| Leirion | — | 195 | Amos | — | 224 |
| Matron | — | 174 | H.C. | — | 24 |
| Isabel | 7 | 557 | H. S. D. | 7 | 7 |
| F. C. J. | — | 305 | | | |

### RESULTS OF SECOND QUARTERLY WORD COMPETITION.

Patience has succeeded in gaining the highest total for dissection of words during the past quarter, but is debarred, according to rule, from receiving a prize, having already gained **two** other prizes during the year. We therefore award the

First Prize (15s.) to Nurse Duty (Miss A. Slaughter, Royal Hospital, Putney).

Second Prize, a tie between Esperance (Miss E. Crowley, 3, Dawson-terrace, Haverstock Hill) and M. W. (Miss Woodward, 123, New Bond-street). We therefore combine the **second and third prizes**, and award 7s. 6d. to each.

### A New Year's Gift.

The minster bells were tolling a death-knell for the parting year. Many were listening for those sounds, reminding them that another twelvemonth had passed away. The young and happy heard them merely with careless, half attention; those of graver temperament, with a fuller realisation of what this yearly custom is symbolical.

The solemn strokes fell mournfully enough upon the ear of the young assistant organist of Westerleigh Cathedral, as he sat alone by an expiring fire, fitfully committing to paper the ideas with which his fevered brain was teeming.

A stir and movement overhead told of the mirth and gaiety this world contains for many. Happy laughter, strains of music, from time to time reminded the occupant of the ground floor that the rooms above him were let to the *prima donna* who was to sing to-morrow at a concert, to which all the neighbourhood of Westerleigh looked forward as one of the greatest events of the season.

"It's a thousand pities, Mr. Heriot, sir, that you don't get someone to mention your name to Madame Cantana," the good-natured landlady had said to her lodger. "She's the kindest-hearted artiste I ever come across. Now, I daresay, if I was to tell her ——"

But a sensitive pride, a dread of being deemed intrusive, made the young musician coldly decline to seek an introduction to the celebrated singer.

He ran his eye over a half-finished score. No error struck him, but the notes seemed to dance about, the lines grew unsteady, and a shivering seized him, as he realised that the fire was very low, and the night was becoming colder. His untasted supper had been removed an hour ago; he had no appetite. He was weary, but sleep constantly delayed to come to his refreshment. Madame Cantana's friends would remain to see the new year in, and till the house was quiet it was no use to go to bed.

Lighting the MS. he held in his hand, Alfred Heriot dropped it leaf by leaf upon the blackened embers in the grate, which still retained sufficient life to be rekindled by the sudden blaze. He stretched out his hands towards the welcome heat; the aspect of the room was brighter; the cathedral clock was chiming for midnight. Ere its twelve slow strokes were ended, other compositions had gone to feed the flames; the young man's glance was directed towards the song lying on his desk, the ink still wet upon the paper, the words he had chosen to set to music reverberating in his brain as the glad bells pealed clamorously to greet the year that was just born.

The *prima donna*, whose popularity enabled her to gather around her a circle of admirers in almost every town of the civilised world, informed her visitors, as she bade them adieu, "that she meant to begin this year well, and as a proof of it intended to be in bed by one o'clock."

"There is something rather sad to me," she said to her husband, after the last guest had departed, "in thinking of this season of the year and its old customs. I have everything in life to make me happy, and the longer I live the more enjoyment I receive; but it is a sad, a wretched world for many people. I wish I knew some way to bestow a little true happiness on somebody."

"Carina! As though you did not bestow happiness and delight on thousands of people every hour of your life," exclaimed her husband, gallantly.

Before she could reply to this further than by a smile, there was a tap at the door. The landlady, with many apologies for intruding, had come to beg for a little of the wine madame's visitors had left.

"I've not a drop of anything myself, ma'am," said Mrs. Wilson; "the little brandy I generally keep by me in case of sickness having gone into the Christmas plum pudding, or I would not make so bold. But I can't bring the poor lad to, he's that weak and exhausted."

Very little explanation was needed ere the singer comprehended that a young musician, who was breaking his heart through disappointment and ill-luck, lay in a fainting fit in the room below. She followed the landlady to the chamber. There were tears in her eyes, and in her heart a warmer emotion of gratitude than that feeling she had so recently spoken of to her husband.

It seemed to Alfred Heriot, after he had lost consciousness for a while, that he was dreaming a beautiful dream, as he lay without power of movement in his bed. Through the open door of the adjoining room, where the fire was still alight, he could see the table at which he had been writing, and over that table a spirit form appeared to bend, whilst upon his soothed and contented ear, in softly murmured accents, fell snatches of the melody with which his thoughts had been occupied at the moment when that strange giddiness had over-mastered him. And when morning came he scarcely liked to wake, knowing how those sweet illusions must lose themselves in the cold reality of existence.

He went in good time to the Town Hall, where the concert was to be held, and chose his seat with the view of being removed from any acquaintances, whose criticisms or conversation might disturb his enjoyment. He was consequently a little annoyed when someone, a total stranger to him, taking the next chair, wished him "Good-evening," and observed that "they had a capital programme before them."

Alfred answered courteously, but evinced no inclination to talk. The other, an elderly man, speaking with a foreign accent, did not persist in his essay at sociability, and little or nothing more passed between them until the moment arrived when Madame Cantana was to sing her first song. Then the stranger turned to his neighbour, and asked in a tone of much concern:

"Do you feel ill? You look so pale."

"No, no!" the young man answered, impatiently, whilst a storm of clapping greeted the appearance of the popular cantatrice, drowning his reply. But despite his denial, he was conscious of a strange inexplicable sensation, and though he was not faint, he wondered what it was that caused him suddenly to think of those wild fancies and the quickly passing dreams which had followed his attack of the night before.

The white-robed form of the *prima donna* stood erect and calm in the middle of the platform. The accompanyist struck merely a few chords upon the piano, and then the grand clear voice poured out its soul-entrancing introduction:

> "Ring out the old, ring in the new,
> Ring, happy bells, across the snow;
> The year is going, let him go,
> Ring out the false, ring in the true."

The well-known words were as appropriate to the season as the aspect of the beautiful songstress, who, in her glistening raiment of frost-flecked white, with garlands of snowdrops, looked like a poetical embodiment of New Year. The acclamations with which she had been received were as nothing in comparison with the enthusiasm at the conclusion of the song. Surely he must be dreaming now, the young composer thought, but was it another blissful hallucination, or the most cruel nightmare, from which he would awaken with a sob of bitter sorrow? Had someone else conceived the same idea before him? Did the notes ever arrange themselves precisely in the same way in two separate brains? Had there been a shameful, wanton robbery? He remembered having destroyed much of his music, but he thought that last completed work was still lying on his open desk.

"Madame Cantana would like you to accompany her in your song this time. She determined it should be encored. That is why I wished to make sure of finding you, that I might have the pleasure of conducting you to the orchestra."

So said the good friend at Alfred's side, as taking him by the arm he drew him from the hall. The young man was too much dazed and bewildered to ask what it all meant, his guide in too great a hurry to waste breath upon any information.

A speedy transit brought them to the room where the different artists awaited their turns to perform. Here was Madame Cantana, a flush on her fair face, her eyes bright with tears of pleasure.

"You are not angry at this little stratagem?" she whispered, as she took the young musician's hand to lead him towards the platform. "It was a whim of mine to make you a new year's gift, and whilst you were asleep I took a copy of the music. I was in despair lest you should be too ill to go out to-day, and that I should be unable to study it properly without you hearing me. This song is charming, and shall make your fame. I will sing it at every concert whilst this new year is young, and everyone shall ask for copies of it until the publishers are unable to provide them quickly enough, and become wild to obtain more works from the same composer."

